# Early clinical outcomes of on-pump beating-heart versus off-pump technique for surgical revascularization in patients with severe left ventricular dysfunction: the experience of a single center

**DOI:** 10.1186/s13019-017-0572-x

**Published:** 2017-02-23

**Authors:** LiMin Xia, Qiang Ji, Kai Song, JinQiang Shen, YunQing Shi, RunHua Ma, WenJun Ding, ChunSheng Wang

**Affiliations:** 1 Shanghai Cardiovascular Disease Research Institute, 180 Fenglin Rd., Shanghai, 200032 People’s Republic of China; 2 Department of Cardiovascular Surgery of Zhongshan Hospital Fudan University, 1609 XieTu Rd., Shanghai, 200032 People’s Republic of China

**Keywords:** Coronary artery bypass grafting, Severe left ventricular dysfunction, On-pump beating heart surgery, Off-pump

## Abstract

**Background:**

Limited experiences of applying an on-pump beating-heart technique for surgical revascularization in patients with severe left ventricular dysfunction have been reported. Which strategy, either off-pump coronary artery bypass grafting (CABG) or on-pump beating-heart CABG surgery, is the best strategy for surgical revascularization in patients with severe left ventricular dysfunction is still controversial. This single-center study aimed to evaluate the impacts of an on-pump beating-heart versus an off-pump technique for surgical revascularization on the early clinical outcomes in patients with a left ventricular ejection fraction (LVEF) of 35% or less to explore which technique would be more suitable for surgical revascularization in patients with severe left ventricular dysfunction.

**Methods:**

A total of 216 consecutive patients with an echocardiographic estimated LVEF of 35% or less who underwent non-emergency, primary, isolated CABG from January 2010 to December 2014 were included in this study and were divided into either an ONBEAT group (patients who received on-pump beating-heart CABG surgery, *n* = 88) or an OFF group (patients who received off-pump CABG surgery, *n* = 128). The early clinical outcomes were investigated and compared.

**Results:**

Patients in the ONBEAT group compared to the OFF group had a significant higher early postoperative LVEF (35.6 ± 2.9 vs. 34.8 ± 3.3%, *p* = 0.034) but shared a similar baseline LVEF (31.0 ± 2.8 vs. 31.0 ± 2.9%, *p* = 0.930). Patients in the ONBEAT group compared to the OFF group received a greater number of grafts and an increased amount of drainage during the first 24 h (3.7 ± 0.8 vs. 2.8 ± 0.6, *p* <0.001; 715 ± 187 ml vs. 520 ± 148 ml, *p* <0.001, respectively), without evidence of worse in-hospital mortality or major postoperative morbidity. Additionally, logistic regression analysis showed that surgical technique (on-pump beating-heart CABG vs. off-pump CABG) had no independent influence on in-hospital mortality or major postoperative morbidity in patients with preoperative LVEF of 35% or less.

**Conclusions:**

The on-pump beating-heart technique may be an acceptable alternative to the off-pump technique for surgical revascularization in patients with an estimated LVEF of 35% or less.

## Background

With advances in instruments, myocardial protection and surgical technology, both early and long-term outcomes of surgical revascularization are increasingly improved. Consequently, a growing number of high-risk patients suffering from coronary artery disease are expected to be treated with surgical revascularization [1–5].

Off-pump coronary artery bypass grafting (CABG), which avoids the use of cardiopulmonary bypass with cardioplegic arrest and aortic cross-clamp, has attracted the interest of an increasing number of cardiac surgeons and patients scheduled for surgical revascularization [6, 7]. Although most proponents of off-pump surgery have advocated the off-pump technique as a suitable strategy for surgical revascularization, patients with severe left ventricular dysfunction undergoing off-pump CABG surgery are at risk for intra-operative hemodynamic deterioration and incomplete revascularization, which increases the incidence of postoperative morbidity and mortality [8]. Even if a combined application of an intra-aortic balloon pump (IABP), morbidity and mortality after off-pump CABG surgery may remain high in patients with severe left ventricular dysfunction due to intra-operative hemodynamic deterioration resulting from inadequate circulatory support (IABP support increases the cardiac output up to approximately 17% in the case of severe pump failure) [9, 10].

On-pump beating-heart CABG, which uses cardiopulmonary bypass without either cardioplegic arrest or aortic cross-clamp, has recently been applied in high-risk patients [11–19]. Cardiopulmonary bypass offers enough circulatory support and ensures intra-operative hemodynamic stability more than IABP support does. Assisted by cardiopulmonary bypass, the optimal exposure of the target coronary artery can be obtained due to perfect cardiac decompression, which contributes to complete revascularization. However, despite avoiding the disadvantages resulting from cardioplegic arrest and aortic cross-clamp, on-pump beating-heart CABG is associated with postoperative morbidity related to the use of cardiopulmonary bypass [15]. Additionally, limited experiences of applying an on-pump beating-heart technique for surgical revascularization in high-risk patients with severe left ventricular dysfunction have been reported.

Which strategy, either off-pump CABG surgery or on-pump beating-heart CABG surgery, is the best strategy for surgical revascularization in patients with severe left ventricular dysfunction is still controversial. Few reports have focused on evaluating the impacts of an on-pump beating-heart versus an off-pump technique for surgical revascularization on the clinical outcomes in patients with a left ventricular ejection fraction (LVEF) of 35% or less. This single-center, retrospective study reviewed 216 consecutive patients with an echocardiographic estimated LVEF of 35% or less who underwent non-emergency, primary, isolated CABG surgery, and evaluated the impacts of the on-pump beating-heart approach versus the off-pump technique on the early clinical outcomes to explore which technique would be more suitable for surgical revascularization in patients with an estimated LVEF of 35% or less.

## Methods

### Patients

Patients undergoing non-emergency, primary, isolated CABG surgery with graftable double- or triple- vessel disease and severe left ventricular dysfunction were eligible for this study. In this study, severe left ventricular dysfunction was defined an echocardiographic estimated LVEF of 35% or less using a modified *Simpson* method. Exclusion criteria included concomitant left ventricle aneurysm, concomitant post-infarction ventricular septal defect, concomitant medium to severe mitral regurgitation or aortic regurgitation, and concomitant acquired or congenital cardiac or aortic surgery.

All included patients were allocated to either an ONBEAT group (patients received on-pump beating-heart CABG surgery) or an OFF group (patients underwent off-pump CABG surgery). The decision to perform on-pump beating-heart or off-pump CABG was influenced by each patient’s demographic and clinical profile (i.e., age, diabetes mellitus, renal function, left ventricular endo-diastolic diameter, and estimated surgical risks), but the choice was ultimately left to the discretion of the operating surgeon. In our center, off-pump CABG surgery was performed routinely for over 10 years before the launch of the trial, and all surgical revascularization procedures in high-risk patients were performed by 3 surgeons who were highly experienced in both off-pump and on-pump CABG surgery (each of the 3 surgeons performed at least 50% of their CABG procedures as off-pump CABG with an annual CABG procedure volume of over 150 cases). Patients were regularly followed up at 1 and 6 months after discharge.

### Surgical procedures

All patients underwent CABG through a median full-sternotomy. The in situ left internal mammary artery, which was skeletonized or dissected as a pedicle according to the surgeon’s preference, was always preferred as the first choice for revascularization of the left anterior descending coronary territory whenever feasible. Saphenous vein grafts and radial arteries were harvested with an open technique. The choices of using grafting conduits and employing a sequential technique for the secondary target vessels were affected by the target coronary territories (i.e., right coronary and left circumflex), graft conduit availability, and the surgeon’s preference concerning these factors for achieving complete revascularization. An eNclose (Novare Surgical Systems Inc., American) or even “no-touch” aorta technique was available when moderate to severe ascending aortic sclerosis or calcification was detected. The quality of anastomosis was assessed after grafting with a transit-time flow probe (Medistim Butterfly Flow Meter, Oslo, Norway).

For patients receiving off-pump CABG, heparin was given to reach an ACT of more than 300 s. The central temperature was maintained above 36 °C to avoid hypothermia-induced ventricular arrhythmia. The heart was displaced using a posterior pericardial stitch and large (12 × 70 cm) gauze swabs. Patients lacking good presentation of the target arteries on the lateral and inferior aspect of the heart were placed in a gentle right decubitus Trendelenburg position to assist in visualization. Stablization of the target coronary arteries was accomplished with a tissue stabilizer (Octopus, Medtronic Corporation, Minneapolis, MN). Meanwhile, medical reduction of heart rate and myocardial contractility was performed with a short-acting beta-blocker. A CO_2_-blower/NaCl mister device was used in situations in which a bloodless field was not achieved with proximal target vessel occlusion. An intra-coronary shunt (Medtronic Corporation, Minneapolis, MN) was used during grafting when necessary. Blood loss was collected with a cell salvage device, and the salvaged blood was re-infused into the patient before completion of the surgery.

For patients undergoing on-pump beating-heart CABG, cardiopulmonary bypass was instituted by cannulating the ascending aorta and right atrium after systemic heparinization (3 mg/kg) with a target activated clotting time of more than 480 s without either cardioplegic arrest or an aortic cross-clamp. Moderate hemodilution (hematocrit, 20 to 25%) and no cooling were used during cardiopulmonary bypass. Standard management included membrane oxygenators, arterial catheter filters, and a non-pulsatile flow of 2.4 L/min/m^2^ with a mean arterial blood pressure greater than 50 mmHg. The same exposure, stabilization, and immobilization techniques to allow exposure of the lateral, posterior, and inferior walls of the heart used during off-pump CABG were applied. A CO_2_-blower/NaCl mister device and an intra-coronary shunt were used during grafting when necessary. After the cardiopulmonary bypass was discontinued, heparin was neutralized with 1 mg protamine sulfate per 1 mg provided.

### End point

The primary end point was in-hospital mortality, which was defined as death occurring during the hospitalization at which the operation was performed, even if death occurred after 30 days or after discharge from the hospital but within 30 days of the procedure, unless the cause of death was clearly unrelated to the operation.

The secondary end point was the occurrence of major postoperative morbidity, including circulatory morbidity (i.e., low cardiac output syndrome (LCOS) and new onset of acute myocardial infarction), pulmonary complications (i.e., pneumonia and respiratory failure), renal failure requiring hemodialysis, stroke, re-operation for bleeding, and deep sternal wound infection. Postoperative LCOS was defined as the requirement for IABP and/or inotropic support for inability to discontinue cardiopulmonary bypass or for longer than 30 min after the patient was returned to intensive care unit to maintain the systolic blood pressure >90 mmHg and the cardiac index >2.2 L/min/m^2^ [20]. New onset of acute myocardial infarction after the operation was defined any new Q wave or disappearance of an R wave on a postoperative electrocardiogram within 24 h of the operation [21]. Postoperative pneumonia was defined as a positive result in a sputum culture requiring anti-infection treatment, or a chest X-ray diagnosing pneumonia following cardiac surgery. Postoperative respiratory failure was defined as a duration of mechanical ventilation for more than 72 h or re-intubation following cardiac surgery. Postoperative stroke was defined as a new focal or global dysfunction of cerebral function lasting over 24 h. Deep sternal wound infections (bone-related or any drainage of purulent material from the sternotomy wound and instability of the sternum) were recorded. In addition, the length of ICU stay, the amount of drainage during the first 24 h, the duration of mechanical ventilation, and IABP support (prophylactic preoperative application and application on an “as needed” basis) were also recorded. The decision to prophylactic preoperative IABP application was influenced by each patient’s demographic and clinical profile (i.e. estimated surgical risks, hemodynamic instability before grafting, etc.), but the choice was ultimately left to the discretion of the operating surgeon. Patients were installed with an IABP when they were inability to discontinue cardiopulmonary bypass after grafting or developed low cardiac output after CABG surgery (application of IABP on an “as needed” basis).

### Statistical analysis

This was a single-center, retrospective study. This study protocol was approved by the ethics committee of Zhongshan Hospital Fudan University and was consistent with the *Declaration of Helsinki*. Peri-operative data were obtained from our institutional database and reviewed using a standard data collection form.

Normally distributed continuous variables were expressed as the mean ± standard deviation and were compared between groups using the *Student’s t*-test. Non-normally distributed continuous variables were expressed as median and quartiles and were compared between the groups with the *Wilcoxon rank sum* test. Categorical variables were expressed as frequency distributions and single percentages, and they were compared between groups using *χ* ^*2*^ test and *Fisher’s* exact test, when appropriate. An in-hospital survival analysis was conducted using the Kaplan-Meier method with a log-rank test for group comparisons. To further confirm the reliability of the results, in-hospital mortality and major postoperative morbidity were further analyzed using a multivariate logistic regression analysis. The covariate of regression analysis included age, gender, smoking, past medical history (including diabetes, hyperlipemia, hypertension, chronic obstructive pulmonary disease, chronic renal dysfunction, cerebro-vascular accident, myocardial infarction, congestive heart failure, and peripheral vascular disease), left ventricular ejection fraction, left ventricular end-diastolic diameter, and the number of grafts. A value of two-sided *p* less than 0.05 was considered statistically significant. Statistical analysis was performed with SPSS statistical package version 17.0 (SPSS Inc., Chicago, IL, USA).

## Results

### Study population

From January 2010 to December 2014, 257 patients with double- or triple- vessel coronary artery disease met the inclusion criteria and accounted for 7.0% of all patients with coronary artery disease who received CABG surgery. Forty-one patients were excluded due to a concomitant left ventricle aneurysm in 20 patients, a concomitant medium to severe mitral regurgitation or aortic regurgitation in 18 patients, and a concomitant post-infarction ventricular septal defect in 3 patients. Finally, 216 patients (45 females, mean 65.5 ± 8.1 years of age) were analyzed with their LVEF varying from 18–35% (31.0 ± 2.9%). Among these patients, 88 patients who received on-pump beating-heart CABG were included in the ONBEAT group, and the remaining 128 patients who received off-pump CABG were included in the OFF group.

The baseline characteristics of the entire cohort are shown in Table 1. Patients in the ONBEAT group were more likely to present with diabetes and a larger left ventricular endo-diastolic diameter, and they had a higher proportion of enlarged left ventricles compared to the OFF group (37.5 vs. 24.2%, *p* = 0.048; 66.8 ± 6.0 mm vs. 64.8 ± 5.6 mm, *p* = 0.013; and 56.8 vs. 39.8%, *p* = 0.018, respectively). Eighteen patients in the OFF group received prophylactic preoperative IABP support, whereas no patients in the ONBEAT group received prophylactic preoperative IABP support (*p* <0.001). No significant differences emerged between the 2 groups in age and the proportions of older age, gender, recent smoking, chronic obstructive pulmonary disease, hypertension, diabetes, hyperlipemia, chronic renal dysfunction, prior cerebro-vascular accident, history of percutaneous coronary intervention, recent myocardial infarction, congestive heart failure, the extent of coronary artery disease, left main coronary artery disease, left ventricular ejection fraction, and a Euro-SCORE >6.0.

**Table 1:** Baseline and operative characteristics of the entire cohort

	Group ONBEAT (*n* = 88)	Group OFF (*n* = 128)	*p*
Preoperation			
Age (years)	66.0 ± 7.9	65.2 ± 8.2	0.509
Older age (age >65 years)	54 (61.4%)	75 (58.6%)	0.778
Gender (Female)	18 (20.5%)	27 (21.1%)	1.000
Recent smoking	17 (19.3%)	21 (16.4%)	0.859
COPD	11 (12.5%)	17 (13.3%)	1.000
Hypertension	48 (54.5%)	73 (57.0%)	0.781
Diabetes	33 (37.5%)	31 (24.2%)	0.048
Hyperlipemia	13 (14.8%)	19 (14.8%)	1.000
Chronic renal dysfunction	2 (2.3%)	5 (3.9%)	0.703
Prior CVA	7 (8.0%)	17 (13.3%)	0.274
History of PCI	16 (18.2%)	24 (18.8%)	1.000
Recent MI	22 (25.0%)	28 (21.9%)	0.625
Congestive heart failure	25 (28.4%)	38 (29.7%)	0.879
Extent of CAD			
2 vessel	4 (4.5%)	12 (9.4%)	0.290
3 vessel	84 (95.5%)	116 (90.6%)	
LM	26 (29.5%)	33 (25.8%)	0.641
LVEF (%)	31.0 ± 2.8	31.0 ± 2.9	0.930
LVEDD (mm)	66.8 ± 6.0	64.7 ± 5.9	0.013
Enlarged left ventricles (LVEDD > 65 mm)	50 (56.8%)	51 (39.8%)	0.018
Prophylactic IABP support	0	18 (14.1%)	<0.001
Euro-SCORE >6.0	44 (50.0%)	61 (47.7%)	0.782
Intra-operation			
Number of grafts	3.7 ± 0.8	2.8 ± 0.6	<0.001
CPB time (min)	83.7 ± 7.7	0	<0.001
Urgent switch to on-pump	0	5 (3.9%)	0.081

*Group ONBEAT* patients received on-pump beating-heart coronary artery bypass grafting, *Group OFF* patients underwent off-pump coronary artery bypass grafting, *COPD* chronic obstructive pulmonary disease, *CVA* cerebro-vascular accident, *PCI* percutanous coronary intervention, *MI* myocardial infarction, *CAD* coronary artery disease, *LM* left main trunk disease, *LVEF* left ventricular ejection fraction, *LVEDD* left ventricular end-diastolic diameter, *IABP* intra-aortic balloon pump, *Euro-SCORE* European system for cardiac operative risk evaluation, *CPB* cardiopulmonary bypass

### Intra-operative data

The number of bypass conduits ranged from 2 to 6. The left internal mammary artery was used as a bypass conduit in 216 patients (100%), the right internal mammary artery was used as a graft for 70 patients (32.4%), a radial artery graft was used for 32 patients (14.8%) and a saphenous vein graft was used for 198 patients (91.7%). Patients in the ONBEAT group compared to the OFF group received a greater number of grafts (3.7 ± 0.8 vs. 2.8 ± 0.6, *p* = 0.000). The duration of cardiopulmonary bypass in the ONBEAT group was 83.7 ± 7.7 min. Five patients (3.9%) scheduled for off-pump CABG surgery had to be switched urgently to on-pump beating-heart CABG surgery due to hemodynamic deterioration or frequent ventricular fibrillation during the operation.

### Preoperative and early postoperative LVEF

Mean LVEF, as measured preoperatively and early postoperatively (before discharge), significantly improved from 31.0 ± 2.8 to 35.6 ± 2.9% (*p* <0.001) in the ONBEAT group and from 31.0 ± 2.9 to 34.8 ± 3.3% (*p* <0.001) in the OFF group, respectively. Patients in the ONBEAT group compared to the OFF group had a significant higher early postoperative LVEF (35.6 ± 2.9 versus 34.8 ± 3.3%, *p* = 0.034) but shared a similar baseline LVEF (31.0 ± 2.8 versus 31.0 ± 2.9%, *p* = 0.930).

### In-hospital mortality and major postoperative morbidity

Nine patients died during the same hospitalization or within 30 days of the operation, and the in-hospital mortality was 4.2%. The causes of death were as follows: low cardiac output in 4 patients, infection in 2 patients, malignant arrhythmia in 1 patient, cerebral hemorrhage in 1 patient, and gastrointestinal bleeding in 1 patient. Patients in the ONBEAT group compared to the OFF group had a slightly lower in-hospital mortality, but no significant difference was found (3.4 vs. 4.7%, *p* = 0.741). Additionally, 2 patients out of 5 patients undergoing urgent switching from off-pump to on-pump beating-heart CABG surgery died as follows: one died of postoperative low cardiac output, and the other died of malignant arrhythmia.

The major postoperative morbidity is summarized in Table 2. Patients in the ONBEAT group compared to the OFF group had a low circulatory morbidity, including low incidences of postoperative low cardiac output syndrome and new onset of acute myocardial infarction, but no significant differences were found (12.5 vs. 19.5%, *p* = 0.197; 3.4 vs. 3.9%, *p* = 1.000, respectively). There was no significant difference in the proportion of application of IABP support on an “as needed” basis between the 2 groups (12.5 vs. 19.5%, *p* = 0.197). After an IABP insertion, 4 patients out of 54 patients developed either limb ischemia or hematoma at the site of IABP insertion. All 4 patients recovered after discontinuation of IABP. There were no IABP-related deaths.

**Table 2:** Early postoperative outcomes

	Group ONBEAT (*n* = 88)	Group OFF (*n* = 128)	*p*
In-hospital mortality	3 (3.4%)	6 (4.7%)	0.741
Circulatory morbidity			
LCOS	11 (12.5%)	25 (19.5%)	0.197
New onset of acute MI	3 (3.4%)	5 (3.9%)	1.000
IABP support on an “as needed” basis	11 (12.5%)	25 (19.5%)	0.197
Drainage during the first 24 h (ml)	715 ± 187	520 ± 148	0.000
Re-operation for bleeding	3 (3.4%)	3 (2.3%)	0.689
Pulmonary complications			
Pneumonia	10 (11.4%)	12 (9.4%)	0.653
Respiratory failure	14 (15.9%)	11 (8.6%)	0.129
Duration of MV (h, median)	29	28	0.483
Renal failure requiring hemodialysis	3 (3.4%)	4 (3.1%)	1.000
Stroke	5 (5.7%)	5 (3.9%)	0.533
Deep sternal wound infection	4 (4.5%)	5 (3.9%)	1.000
Length of ICU stay (d)	4.0 ± 3.1	3.9 ± 1.9	0.517
LVEF before discharge (%)	35.6 ± 3.1	34.8 ± 3.3	0.034

*Group ONBEAT* patients received on-pump beating-heart coronary artery bypass grafting, *Group OFF* patients underwent off-pump coronary artery bypass grafting, *LCOS* low cardiac output syndrome, *MI* myocardial infarction, *IABP* intra-aortic balloon pump, *MV* mechanic ventilation, *ICU* intensive care unit, *LVEF* left ventricular ejection fraction

Patients in the ONBEAT group compared to the OFF group had a slightly higher incidence of postoperative pulmonary complications, including slightly higher incidences of pneumonia and respiratory failure, but no significant differences emerged (11.4 vs. 9.4%, *p* = 0.653; 15.9 vs. 8.6%, *p* = 0.129, respectively). Patients in the ONBEAT group compared to the OFF group had prolonged mechanical ventilation, but no significant differences emerged (median, 29 vs. 28 h, *p* = 0.483).

Patients in the ONBEAT group compared to the OFF group had a low incidence of renal failure requiring hemodialysis and high incidences of stroke and deep sternal wound infection, but no significant differences emerged. Despite an significantly increased amount of drainage during the first 24 h (715 ± 187 vs. 520 ± 148 ml, *p* <0.001), patients in the ONBEAT group had a similar incidence of re-operation for bleeding compared to the OFF group (3.4 vs. 3.1%, *p* >0.05). Additionally, the 2 groups had a similar length of ICU stay (4.0 ± 3.1 vs. 3.9 ± 1.9 d, *p* = 0.517).

The impacts of surgical technique (on-pump beating-heart CABG vs. off-pump CABG) on in-hospital mortality and major postoperative morbidity after adjusting for potential confounders (i.e., age, gender, smoking, diabetes, hyperlipemia, hypertension, chronic obstructive pulmonary disease, chronic renal dysfunction, cerebro-vascular accident, myocardial infarction, congestive heart failure, left ventricular ejection fraction, left ventricular end-diastolic diameter, and the number of grafts) are shown in Table 3. In multivariate logistic regression analysis, surgical technique (on-pump beating-heart CABG vs. off-pump CABG) had no independent influence on in-hospital mortality or major postoperative morbidity.

**Table 3:** Impacts of surgical techniques on mortality and morbidity

Events	Group OFF	Group ONBEAT
In-hospital mortality	1.0	0.75 (0.38–2.16)
LCOS	1.0	0.61 (0.31–1.86)
New onset of acute MI	1.0	0.65 (0.34–2.05)
Pneumonia	1.0	1.31 (0.72–3.85)
Respiratory failure	1.0	1.75 (0.83–4.86)
Renal failure	1.0	0.78 (0.41–2.24)
Stroke	1.0	2.18 (0.93–5.79)
Re-operation for bleeding	1.0	1.11 (0.58–3.25)
Deep sternal wound infection	1.0	1.25 (0.63–3.84)
IABP support on an “as needed” basis	1.0	0.62 (0.33–1.98)

*Group OFF* patients underwent off-pump coronary artery bypass grafting, *Group ONBEAT* patients received on-pump beating-heart coronary artery bypass grafting, *LCOS* low cardiac output syndrome, *MI* myocardial infarction, *IABP* intra-aortic balloon pump

## Discussion

The major finding of the current study was that patients in the ONBEAT group compared to the OFF group received an increased amount of drainage during the first 24 h, a greater number of grafts, and a more improved LVEF, without evidence of worse in-hospital mortality or major postoperative morbidity. In this study, patients in the ONBEAT group compared to the OFF group received an increased amount of drainage during the first 24 h. The reason may be related to cardiopulmonary bypass and heparinization during on-pump beating-heart CABG surgery. Although an increased amount of drainage occurred during the first 24 h, the 2 groups shared a similar incidence of re-operation for bleeding. Patients in the ONBEAT group compared to the OFF group received a greater number of grafts, which may have been be result of optimal exposure of the target coronary artery due to perfect cardiac decompression with the support of cardiopulmonary bypass. Patients in the ONBEAT group compared to the OFF group had a significant higher early postoperative LVEF but shared a similar baseline LVEF, suggesting that patients in the ONBEAT group received a more improved LVEF after CABG surgery. It meant that patients with severe left ventricular dysfunction may have more benefits from on-pump beating-heart CABG surgery. The reason may be related to a greater number of grafts in the ONBEAT group compared to the OFF group. Although there was no significant difference, patients in the ONBEAT group compared to the OFF group had a slightly low incidence of circulatory morbidity. The reasons may be that the surgical technique and cardiopulmonary bypass can offer enough circulatory support and ensure hemodynamic stability during on-pump beating-heart CABG surgery. A low incidence of circulatory morbidity may also be related to complete revascularization, which was revealed in this study when patients in the ONBEAT group received a greater number of grafts compared to the OFF group. Although there was no significant difference, patients in the ONBEAT group compared to the OFF group had a slightly higher incidence of postoperative pulmonary complications. The reason for high incidence of pulmonary complications may be related to the application of extracorporeal circulation during on-pump beating-heart surgery.

Previous studies have investigated the on-pump beating-heart technique for surgical revascularization. Even by 2005, Rastan and colleagues [17] had prospectively examined randomized data of markers of myocardial injury in 20 patients with a normal ejection fraction who underwent off-pump CABG compared to 20 patients with a normal ejection fraction who underwent on-pump beating-heart CABG, and they showed off-pump CABG had less myocardial injury than on-pump beating-heart CABG. This evidence differed from the results of this study. The reason for this difference may have been the study population because the current study focused on high-risk patients with left ventricular ejection fraction of 35% or less. Gulcan and colleagues [12] evaluated myocardial function and clinical outcomes of 46 high-risk patients with an ejection fraction <30% who received on-pump beating-heart CABG and associated procedures, and they found that the on-pump beating-heart CABG technique was effective for protecting myocardial functions in patients with severe left ventricular dysfunction and was associated with low postoperative morbidity and mortality. This evidence agreed with the outcomes of the current study. Another study [22], including patients with a high-risk Euro-SCORE and with a mean EF of 0.37, revealed that the on-pump beating-heart method seemed to be safe, secure and effective for this population of very high-risk patients and conducive to complete revascularization, reducing early complications and multi-organ failure compared to off-pump CABG. This evidence also agreed with the outcomes of this study. A recent study [23] investigated the early outcomes associated with survival, morbidity and improvement of left ventricular function in 66 high-risk patients with a mean ejection fraction of 0.27 undergoing conventional CABG and 65 high-risk patients with a mean ejection fraction of 0.28 undergoing on-pump beating-heart CABG, and showed that the on-pump beating-heart technique may be an acceptable alternative to the conventional technique due to lower postoperative mortality and morbidity, and the on-pump beating-heart approach was the preferred method for surgical revascularization in high-risk patients with severe left ventricular dysfunction. This evidence was in line with the outcomes of the current study. Additionally, the results obtained by Darwazah [15] suggested that on-pump beating-heart CABG was more effective in high-risk patients with better myocardial revascularization, but it also had a higher incidence of postoperative morbidity and mortality compared to off-pump CABG. This evidence was in line with evidence from this study of better myocardial revascularization but was different from the incidence of postoperative morbidity and mortality.

In this study, patients in the ONBEAT group had a higher proportion of diabetes compared to the OFF group. The reason may be partly related to the relatively small target coronary artery in diabetic patients. In addition to a small target coronary artery, diabetic patients had more serious and diffuse coronary artery disease compared to the non-diabetic patients. On-pump beating-heart CABG compared to off-pump CABG provides optimal exposure of the target coronary artery and may be the preferred choice for diabetics who required surgical revascularization. This study also showed patients in the ONBEAT group compared to the OFF group had a larger left ventricular endo-diastolic diameter and had a higher proportion of enlarged left ventricles, which may be related to the surgical field because the on-pump beating-heart technique provided optimal exposure of the target coronary artery due to perfect cardiac decompression with the support of cardiopulmonary bypass. Patients in the OFF group compared to the ONBEAT group received a higher proportion of prophylactic preoperative IABP application, suggesting that due to the lack of circulatory support, more high-risk patients with severe left ventricular dysfunction who were scheduled for off-pump CABG surgery required prophylactic use of IABP support. Additionally, 5 patients undergoing urgent switching from off pump to on-pump received cardiopulmonary bypass, but they were still allocated to the OFF group, which may produce biased results. However, due to hemodynamic deterioration or frequent ventricular fibrillation during off-pump CABG surgery, they had to be switched urgently to on-pump beating-heart CABG surgery. Finally, 2 patients out of 5 patients died of low cardiac output or malignant arrhythmia in the peri-operative period. Thus, this result suggested that the on-pump beating-heart approach compared to the off-pump approach may be an suitable choice for patients with severe left ventricular dysfunction and unstable hemodynamics during the operation.

This study showed that on-pump beating-heart CABG compared to off-pump CABG had some advantages, including a more improved LVEF, a greater number of grafts, and a slightly lower incidence of postoperative circulatory morbidity. However, on-pump beating-heart CABG had drawbacks associated with cardiopulmonary bypass, including slightly higher incidences of postoperative pulmonary complications and stroke and an increased amount of drainage during the first 24 h. Additionally, this study was only a single-center, retrospective study that involved a small sample size. It was very difficult to develop an algorithm for the use of on-pump beating-heart CABG as a standard procedure for surgical revascularization in patients with severe left ventricular dysfunction. The decision about using an on-pump beating-heart or an off-pump technique for surgical revascularization in patients with severe left ventricular dysfunction should be tailored individually to achieve the greatest clinical benefits for patients. For example, a diabetic patient with a left ventricular endo-diastolic diameter of 70 mm and an echocardiographic estimated LVEF of 25% may have more benefits from on-pump beating-heart CABG surgery, whereas a patient with severe chronic obstructive pulmonary disease and an echocardiographic estimated LVEF of 35% may benefit from off-pump CABG surgery with or without an IABP support.

There were several limitations to this study. First, it was a single center, retrospective clinical observational study that involved a small sample size, which may have influenced the generalizability of the results. A final determination would need a multi-center study involving a larger sample size. Second, because these were high-risk patients with severe left ventricular dysfunction, this patient group may have produced considerable bias. Third, due to a retrospective clinical observational study, the time course of changes in the serum levels of markers of myocardial injury, e.g., cardiac troponin I, were not dynamically monitored. Finally, this study only focused on evaluating the impacts of the on-pump beating-heart technique vs. the off-pump technique for surgical revascularization on the early clinical outcomes in patients with an LVEF of 35% or less. The medium and long term clinical outcomes needed a further observation.

## Conclusion

The on-pump beating-heart technique may be an acceptable alternative to the off-pump technique for surgical revascularization in patients with severe left ventricular dysfunction due to a greater number of grafts and a more improved LVEF after CABG without evidence of worse in-hospital mortality or major postoperative morbidity. Nevertheless, this study was only a single-center, retrospective, non-randomized clinical observational trial that involved a small sample size. Multi-center, controlled and randomized clinical trials involving larger sample sizes are needed.

## Abbreviations

CABG: Coronary artery bypass grafting surgery
CAD: Coronary artery disease
COPD: Chronic obstructive pulmonary disease
CPB: Cardiopulmonary bypass
CVA: Cerebro-vascular accident
Euro-SCORE: European system for cardiac operative risk evaluation
IABP: Intra-aortic balloon pump
ICU: Intensive care unit
LCOS: Low cardiac output syndrome
LM: Left main trunk disease
LVEDD: Left ventricular end-diastolic diameter
LVEF: Left ventricular ejection fraction
MI: Myocardial infarction
MI: Myocardial infarction
MV: Mechanic ventilation
PCI: Percutaneous coronary intervention
